# An auditory display tool for DNA sequence analysis

**DOI:** 10.1186/s12859-017-1632-x

**Published:** 2017-04-24

**Authors:** Mark D. Temple

**Affiliations:** School of Science and Health, Western Sydney University, Campbelltown Campus, Locked Bag 1797, Penrith South DC, NSW 1797 Australia

**Keywords:** Sonification, DNA sequence, Auditory display

## Abstract

**Background:**

DNA Sonification refers to the use of an auditory display to convey the information content of DNA sequence data. Six sonification algorithms are presented that each produce an auditory display. These algorithms are logically designed from the simple through to the more complex. Three of these parse individual nucleotides, nucleotide pairs or codons into musical notes to give rise to 4, 16 or 64 notes, respectively. Codons may also be parsed degenerately into 20 notes with respect to the genetic code. Lastly nucleotide pairs can be parsed as two separate frames or codons can be parsed as three reading frames giving rise to multiple streams of audio.

**Results:**

The most informative sonification algorithm reads the DNA sequence as codons in three reading frames to produce three concurrent streams of audio in an auditory display. This approach is advantageous since start and stop codons in either frame have a direct affect to start or stop the audio in that frame, leaving the other frames unaffected. Using these methods, DNA sequences such as open reading frames or repetitive DNA sequences can be distinguished from one another. These sonification tools are available through a webpage interface in which an input DNA sequence can be processed in real time to produce an auditory display playable directly within the browser. The potential of this approach as an analytical tool is discussed with reference to auditory displays derived from test sequences including simple nucleotide sequences, repetitive DNA sequences and coding or non-coding genes.

**Conclusion:**

This study presents a proof-of-concept that some properties of a DNA sequence can be identified through sonification alone and argues for their inclusion within the toolkit of DNA sequence browsers as an adjunct to existing visual and analytical tools.

**Electronic supplementary material:**

The online version of this article (doi:10.1186/s12859-017-1632-x) contains supplementary material, which is available to authorized users.

## Background

Alan Turing’s Manchester computer build in 1948 had a built in loudspeaker (referred to as a hooter) that could generate taps, clicks and thumps to indicate the progress of programmed routines [1]. The Programmers’ Handbook for this computer states that “some indication of what is going on is given by the rhythm of the clicks that are heard’. This is an early example of computerised sonification to track the real time progress of a complex routine over an extended period of time. Today's computers are highly capable of performing a variety of analytical tasks to identify otherwise hidden patterns in complex data, one such application has been in the field of genomic data analyses. This paper demonstrated that sonification of DNA sequence data, using algorithms based on biological rules, is useful to determining its properties. These algorithms use codons to generate strings of audio that are representative of ribonucleotides synthesised during transcription. For example, DNA motifs from each of three reading frames may each be assigned to a unique sound to produce three concurrent streams of audio. These can be used to distinguish complex DNA sequence data from repetitive data. Additionally, the occurrence of start or stop codons in either reading frame can be used to turn an audio stream on or off, respectively, which allude to the way DNA may be processed within the cell.

DNA sequence data is linear information that is highly amenable to being sonified within a genomic browser to produce audio. During the past decade there has been a phenomenal increase in the accumulation and storage of DNA sequence data [2] and the strong demand to interrogate these data has lead to the development of many high quality and freely available tools within genomic browsers. Presented herein is one further option to enhance DNA sequence analyses through DNA sonification to create a supplementary auditory display within the browser.

To date, auditory displays have not been widely accepted as a complement to standard visualization of DNA sequence data and they are typically considered to be an interesting oddity that contributes little analytical benefit. However, the use of audio to convey biological information [3] suggest that non-speech sounds are useful to identifying trends in gene sequences. The most obvious characteristics of the auditory displays are the patterns and rhythms of the note phrases rather than the identity of each notes per se, despite the direct mapping of sequence features to distinct notes. Furthermore, assigning audio to DNA [4] or protein [5] sequence data has proven useful to identify DNA properties or protein motifs otherwise hidden from the human eye. These prior approaches are typically founded on the principle of mapping either individual bases [6], codons [4] or amino acids [5] to musical notes in a manner inspired by the genetic code or codon usage during translation. Direct linear mapping of 64 codons to individual notes results in a large octave range and this has generally been avoided since this is not considered to be musically appealing. Therefore, additional rules were devised to reduce the octave range [4] and individual codons were mapped to differing numbers of notes to introduce variation in note duration. In another approach, to improve the musicality of protein sequence data, amino acids were mapped to chords and the number of notes was reduced by mapping pairs of amino acids to the same fundamental note [5, 7]. Earlier studies had mapped each base to two consecutive notes [8] in an octave scale, or in a tone range of a fifth with the GC bases keyed low and the AT bases keyed high [6], this mapping had a minimal note range but was to some degree instructive to the nature of the sequence. Additionally, individual base to note mapping was used to make Genoma Music [9] that was further supplemented with rhythmic and melodic insertions.

Other studies have gone some way to validate sonification as a useful analytical tool to represent the molecular properties of amino acid residue [10], the folding of a protein [11], the spectral properties of DNA molecular analyses [12, 13] or to browse RNA structures [14]. More recently, microbial ecology data has been sonified into a jazz bebop musical style by mapping rows of numerical data to chords [15, 16] with the simple aim of engaging non-specialized people with ecology science. Comparative epigenomic data has been sonified to help communicate the importance of methylation in cancer progression [17]. Gene expression data has been used to create musical compositions that may be used to discriminate between biological samples and to characterise differentially expressed genes [18, 19] and Chip-seq data [20]. Other suggest that sonified DNA/RNA sequence data should be given a far more serious consideration for pattern matching and analysis rather than musical composition [21]. Despite the potential of DNA sonification to enhancing DNA sequence analyses, there has been no integration of real time DNA sonification tools within mainstream genomic browsers. Given recent advances in computer power and browser technology, it is pertinent to consider if integration of sonification and auditory display of DNA sequence data would provide a useful adjunct to the suite of tools currently available in genomic browsers.

### A Morse code for DNA sequence interpretation

The challenge of DNA sonification is to convert linear DNA sequence information into human comprehensible sound in a way that has an implied biological context. There have been other interesting and highly regarded interpretations of genetic information as music [22], which clearly cross the divide between science and art. A more challenging approach assumes DNA to be a non-random sequence and takes into account basic chemical or biological properties during sonification. To achieve this it is important to connect the DNA sequence data to the perceptual characteristics of sound [23]. This study presents a proof-of-concept that sonification has the potential to enhance visual display for inspection of DNA sequence data. The sonification tool and the resultant auditory displays, located at http://dnasonification.org, facilitate basic DNA sequence analyses within an internet browser. It may be helpful to think of this approach as a Morse code for DNA sequence interpretation and not music, despite the fact that musical sounds are used during sonification.

**Fig. 1:**
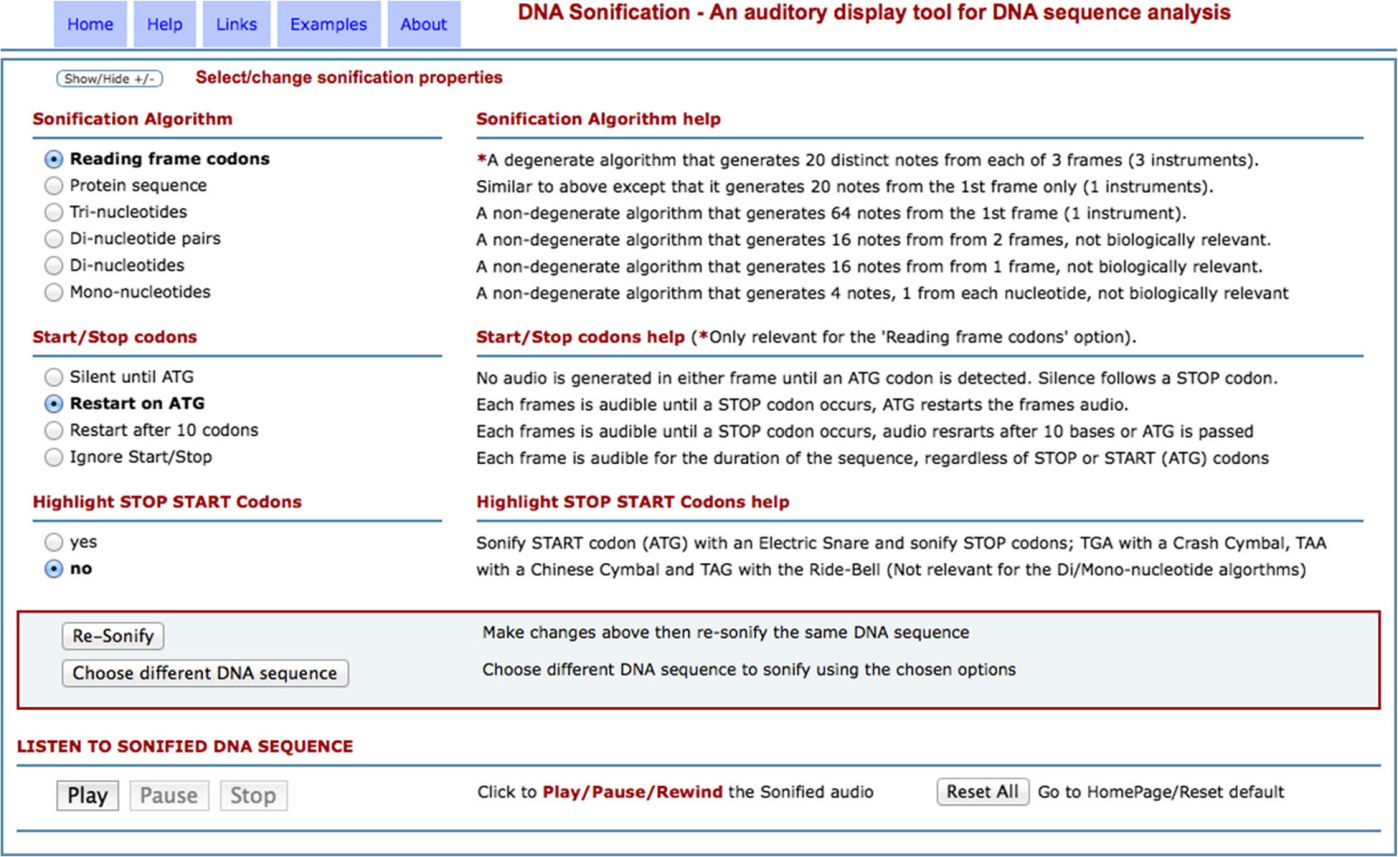
A screen image from the DNA sonification website (http://dnasonification.org). Radio buttons to select either of the six sonification algorithms are located to the left of the page and a brief usage guide to these is located to the right. Below these are options to process start and stop codons and at the bottom left are the controls to play the resultant auditory display

The tool allows users to input their own DNA sequence to generate an auditory display in real time (as shown in Fig. 1), additionally diverse test sequences are provided to highlight the characteristics of each algorithm and resultant auditory display. The main ‘Reading frame codons’ algorithm is faithful to the genetic code whereby each codon is mapped to an individual note in a scale. There is no arbitrary manipulation of this mapping to reduce the number of notes to make the auditory display more musical since this would reduce the discriminatory power of the tool. The bases may be read as codons in each of three possible reading frames leading to three interlaced streams of notes. This approach is advantageous since start and stop codons in either frame have a direct affect to start or stop the audio in that frame, leaving the other frames unaffected. Users can apply different sonification algorithms and change options of these to repeatedly process a DNA sequence to produce a simple or complex auditory display. Hopefully this iterative approach is more informative to an educated user or more interesting to a novice.

## Implementation

Six sonification algorithms were used to map sequential groups of nucleotide bases (DNA motifs) to a range of motif identifier numbers. These numbers were then mapped to specific audio notes to generate an auditory display in the form of a MIDI file playable within a web browser. This format allows for 128 MIDI notes representing approximately 10 octaves. Each algorithm parses DNA sequence in the 5’ to 3’ direction and audio notes are mapped to an arbitrary blues scale (using intervals of 3, 2, 1, 1, 3, 2 in each 12 note octave) in the key of C. In all cases, motif identifier 1 is assigned to the root note of the musical key and the octave is set to match the lowest suitable pitch of the assigned musical instrument. Each algorithm produces a distinct auditory display generated dynamically from the input DNA sequence data which can be played directly in the browser using an audio plugin.

The properties of the six sonification algorithms used on the sonification tools website are summarised in Table 1. The ‘mono-nucleotides’ algorithm simply maps each of four nucleotide bases (G, A, T, or C) to one of only four musical notes. Similarly the ‘di-nucleotides’ algorithm maps pairs of nucleotides as motifs to 16 possible notes. The ‘di-nucleotides pairs’ is a variant of the ‘di-nucleotides’ algorithm, however a second audio stream is generated by advancing the entire sonification frame forward by one base (this is analogous to a second reading frame). The second frame is sonified on a different instrument and consequently twice as many notes are produced (as shown in Table 2). Neither of these three algorithms are sufficient for discriminating between DNA sequence data and are included only for the sake of completeness.

**Table 1:** Summary of the six sonification algorithms and their properties

Algorithm	DNA motif size	Step range (notes)	Number of audio frames (instruments)	Silence on STOP codon Restart on START codon	Sonify occurrence of STOP/START
Reading frame codons	3 bp	20	3	optional	optional
Protein sequence	3 bp	20	1	no	optional
Tri-nucleotides	3 bp	64	1	no	optional
Di-nucleotide pairs	2 bp	16	2	no	no
Di-nucleotides	2 bp	16	1	no	no
Mono-nucleotides	1 bp	4	1	no	no

**Table 2:**
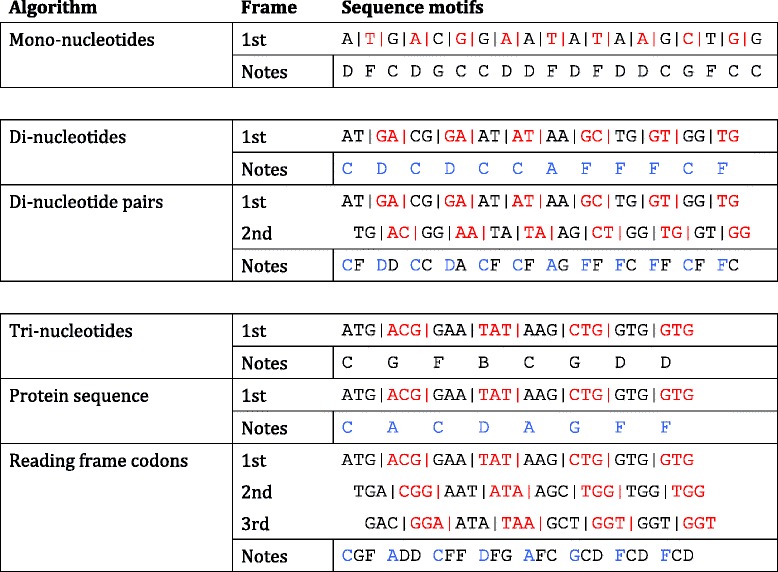
Summary of how a DNA sequence is parsed by each of the six sonification algorithms to produce an auditory display

DNA sequences are coloured red or black to signify adjacent motifs within the reading frame that is parsed by the respective algorithm. Sharps, flats and the octave (pitch) of the notes are not shown for the sake of simplicity. The ‘Di-nucleotide pairs’ algorithm produces a more complex audio display (twice the number of notes) than the ‘Di-nucleotides’ algorithm. Common notes between the two are indicated in blue. Similarly The first ATG codon of the sequence is parsed to a C note by the both the ‘Reading frame codons’ and ‘Protein sequence’ algorithms (coloured in blue). The ‘Reading frame codons’ produces two additional notes absent from the ‘Protein sequence’ algorithm prior to the sonification of the next common codon (ACG) of the first reading frame. The first TGA codon in the second reading frame and the GAC of the third reading frame are parsed to G and F notes, respectively, only by the ‘Reading frame codons’ algorithm

The ‘tri-nucleotides’ algorithm builds upon these algorithms whereby each codon is mapped to one of 64 distinct notes, without degeneracy. Likewise the ‘protein sequence’ algorithm parses the DNA sequential as motifs of three nucleotides (codons) and each of these is mapped to a musical note, however, this algorithm is degenerate since 64 codons are mapped to only 20 notes. This mapping is based on the genetic code in which codons are mapped to only 20 amino acid residues of a protein. Lastly, the ‘Reading frame codons’ algorithm is more discriminatory since all three possible reading frames are sonified. Each reading frame is shifted by one nucleotide and codons of each frame are mapped to one of three distinct sounding instruments. These notes are interlaced into a single audio file as an arpeggiated sequence (also shown in Table 2). In the absence of further processing this gives rise to a characteristic triplet phrasing of notes in the auditory display. The rate of sonification is set to approximately 6 base pairs per second to allow time to perceive each sound. These sonification tools are proposed to supplement visual inspection of short DNA sequences, in the order of a thousand bases in length to create an auditory display of less than 3 min in duration. In all cases the first, second and third frames are mapped to instrument one (piano), instrument two (guitar) and instrument three (organ), respectively. ScoreCloud Studio software was used to convert the MIDI file of the auditory display into musical notation.

### The use of start and stop codons in the sonification algorithms

Additional options are provided for the ‘Reading frame codons’ algorithm to sonify the occurrence of start or stop codons to add more discriminatory power to the audio display. With this algorithm, stop codons cause the audio stream of the reading frame in which they occur to be silenced either until a start codon occurs or until 10 codons have passed. By default the first codons in each reading frame of the DNA sequence produces an audio output unless it is a stop codon, i.e. an ATG start codon is not required to initiate sonification in either reading frame. This default behavior can be circumvented by the ‘Silent until ATG’ option whereby each frame is silent until the occurrence of an in-frame start codon. Additionally the option exists to ignore start stop codons altogether to produce an uninterrupted audio stream representing three reading frames.

Start or stop codons can also be used to trigger distinct audio sounds to highlight their occurrence irrespective of whether the reading frame is silent or not. If this option is selected a potential start codon (ATG) is sonified with a percussive Electric Snare and stop codons are sonified with high frequency cymbal sounds (TGA with a Crash Cymbal, TAA with a Chinese Cymbal and TAG with the Ride-Bell). Sonification of these motifs can be implemented with the ‘Reading frame codons’, ‘Protein sequence’ or ‘Tri-nucleotides’ algorithms (as summarised in Table 1). In this instance the sound is independent of the frame in which it occurs and is additional to the reading frame instrument.

The Sonification tools are implemented through a website interface and whilst the code runs on any browser the ancillary Jazz-Plugin [24] is required to play the in-browser generated MIDI audio file. At the time of writing, this plugin works best in either the Safari, Firefox or Chrome internet browser.

## Results and discussion

Upon first hearing the auditory display of a sonified DNA sequence it may sound like an unfamiliar language or Morse code. If the tool were designed to be musical then more options could be implemented relating to the choice of instrumentation, musical keys, note phrasing, time signatures, chord selection, dynamics, tempo, etc. However, as an analytical tool a more minimal approach is thought to be effective since it does not further abstract the auditory display away from the underlying genetic code, multiple reading frames and functional start and stop codons. To better understand the auditory display, the sonification of a series of test DNA sequences is described, these consist of simple artificial sequences, repetitive sequences or functional DNA sequences. The results from the ‘Reading frame codons’ algorithm are the main focus of this section since it is potentially the most informative as to the nature of the sequence since it is based on the central dogma of molecular biology.

All of the test auditory displays described in this section can be created in real-time using the test and example DNA sequence files available on the sonification tools homepage. Additionally all of these examples have been pre-run and converted into mp3 files (these are available via the ‘Examples’ menu on the homepage at http://dnasonification.org/example.php) since these can be played directly within any browser without the need to install an additional plugin. These auditory display files have also been included as additional files as part of this publication and are discussed below. Each DNA sequence discussed below is listed in Table 1 as ‘Sequence 1’ through to ‘Sequence 11’ In some instances a DNA sequence was sonified with different selected options to highlight how these effect the auditory display.

### Sonification of simple artificial DNA sequences

Sonification of the simple mononucleotide ‘G Sequence’ (shown in Table 3, Sequence 1) is useful to understanding the basic sonification output and it highlights the characteristic triplet note phrasing which underpins the subsequent results (listen to Additional file 1, which is an MP3 copy of this auditory display). Since the same codon motif (GGG) occurs in each reading frame, the same note is played on each of three instruments giving rise to a highly repetitive pattern. The introduction of a point mutation into the ‘G Sequence’ (shown in Table 3, Sequence 2) causes only a transient change of one note in each reading frame/instrument (i.e. a change of up to three notes in one triplet, allowing for degeneracy in the genetic code), as exemplified by a perceivable change in the auditory display of the ‘Mutated G Sequence’ (listen to Additional file 2) at approx. 3 s from the start, no further change is evident. The introduction of stop codons into the ‘G Sequence’ sequence provides further insight into the sonification algorithms and the resultant auditory display. The auditory display of the ‘G Sequence with STOP in the 1st Reading Frame’ (shown in Table 3, Sequence 3) is identical to the ‘G Sequence’ display until the stop codon is parsed (at about 3 s from the start), after which instrument 1 (piano) becomes silent for the remainder of the display (listen to Additional file 3). The stop codon itself (TAA) is silent in frame one (as is the remainder of that frame) whereas in frames two and three the motif manifests as AAG and AGG, respectively, and gives rise to distinct notes before the reoccurrence of the GGG motif (codon). Following the stop codon, and for the remainder of the auditory display, the recurring triplet note phrase is replaced by a recurring two note phrase (with a rest beat in place of the absent instrument). The result of two stop codons in close proximity in different reading frames results in an auditory display of a single instrument with notes separated by two rest beats. Extending this example to include stop codons in all reading frames (shown in Table 3, Sequence 4) result in complete silencing of the remaining auditory display as the third stop codon is parsed (listen to Additional file 4). Re-sonification of this sequence with the ‘Restart after 10 codons’ option additionally forces the audio (Additional file 5) in all frames to become audible after 10 codons (at approx. 9 s), even in the absence of start codons.

**Table 3:**
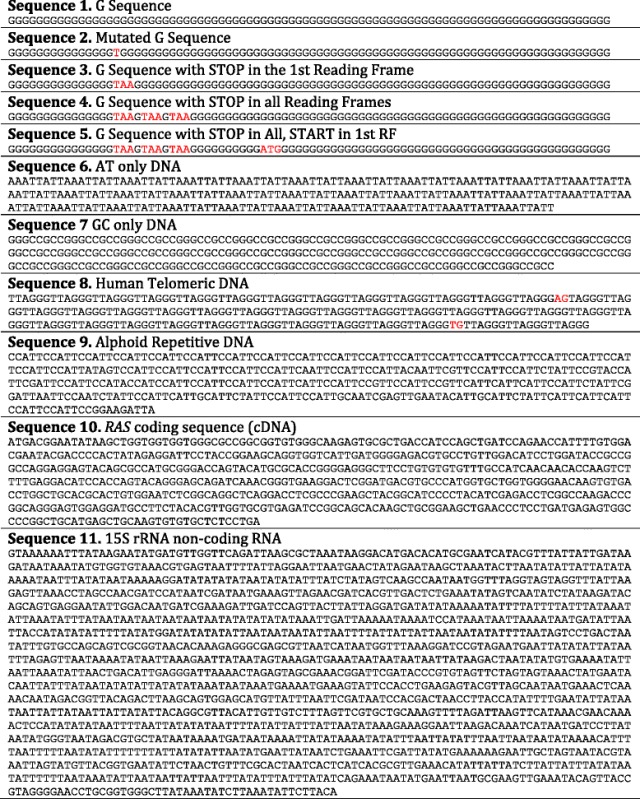
Test input DNA sequences used to assess the DNA sonification algorithms

All sequence names refer to input sequence data available on the DNA sonification homepage. Red text indicates stop and start codons in the ‘G Sequence’ and also sequence variations in the ‘Mutated G Sequence’ and ‘Human Telomeric DNA’ sequence

Due to the importance of stop codons in the control of gene expression, the sonification algorithms provide three options to assess their impact in the auditory display. One option is to silence the audio of the reading frame until the occurrence of an ATG start codon that frame (this is the default setting). This is probably the most informative to asses the transcription potential of the input sequence, however, no account of promoter sequence motifs or the occurrence of ATG as a codon for Methionine are considered at this stage. This simple assumption is consistent with the notion that most intergenic sequences are differentially transcribed [25] and the transcriptome is larger than anticipated by our current understanding of the control of gene expression. Any further discussion of the role of start codons in the role of gene expression is beyond the scope of this study. The second option is to silence the audio for ten codons following a stop codon, this enables the sonification of features of the reading frame further downstream (beyond 30 nucleotides). This option is effective at detecting the occurrence of multiple stop codons in a single frame prior to the occurrence of an ATG. Lastly, stop codons can be simply sonified as a normal codon with out further impact. On the homepage these options are referred to as ‘Restart on ATG’, ‘Restart after 10 codons’ and ‘Ignore Start/Stop’ respectively.

In a manner analogous to the sonification of stop codons, start codons also have a big impact on the sonification algorithm as indicated above and again there are three options for their implementation. In all cases, start codons (or at least the occurrence of an ATG), are mapped to a regular note and treated as a switch to turn on audio from an otherwise silent frame/instrument, if the audio stream is already audible then no change is apparent. The default setting for the sonification tools is the ‘Restart on ATG’ as discussed above. Additionally with this setting all frames are sonified from the beginning of the input sequence, until the occurrence of stop codon. This behaviour can be modified with the ‘Silent until ATG’ option in which case all frames are silent at the initiation of the sonification process until the parsing of a start codon. Lastly, start codons may be sonified as a normal codon with out further impact with the aforementioned ‘Ignore Start/Stop’ option.

A simple example of an ATG as a potential start codon is provided in the sonification of Sequence 5 (Table 3) sonified with the ‘Restart on ATG’ default option. This sequence is similar to Sequence 4 except that following the three stops codons there is a start codon in reading frame 1. The ATG codon causes the audio stream (piano) for the frame in which it occurs to restart at approx. 7 s (listen to Additional file 6), whilst the others frames/instruments remain silent. Audio notes from the solo piano are staggered with two rests beats between each note due to the two silent frames.

### Sonification of repetitive DNA sequence

This section describes the sonification of repetitive DNA sequences. In the first instance the sonification of two synthetic DNA sequences consisting of either A and T or G and C bases are described.

The auditory display of the AT only DNA sequence (see Table 3, Sequence 6) with the default options has the characteristic triplet pattern (three instruments) however due to the high chance of a TAA stop codon in each reading frame the audio becomes silent after only 4 s (Additional file 7) and remains so for the remaining 39 s of the display. The absence of G precludes the occurrence of an ATG codon to restart the audio. Selecting the ‘Restart after 10 codons’ causes no change compared to the default settings due to the re-occurrence of TAA’s (stop codons) within the passage of 10 codons. In contrast the ‘Ignore Start/Stop’ option results in sonification of the entire sequence with the characteristic triplet phrasing (listen to Additional file 8). It may not be obvious but the audio phrasing repeats every 8 triplets due to the repetitive nature of the artificial DNA sequence and this is more apparent with the Tri-nucleotides algorithm that sonifys only the first frame using a wider range of pitch (since this is a non degenerate algorithm).

The auditory display of the GC only DNA sequence (see Table 3, Sequence 7, Additional file 9) plays with the characteristic triplet pattern for the entire 43 s with the default ‘Restart on ATG’ settings or the ‘Ignore Start/Stop’ option. There are no interruptions to the auditory display which is in stark contrast to the display of the AT rich DNA using the same default settings. This is because stop codons (and indeed the start codon) additionally require A and T bases which are absence in the GC DNA. The default ‘Restart on ATG’ setting clearly has a discernably impact on the rhythm and timing of notes in the auditory display of the AT sequence but not the GC sequence. Again the ‘Tri-nucleotides’ algorithm demonstrate that the DNA sequence repeats without variation every 8 codons which is difficult to discern by visual inspection alone. However with this algorithm it is not possible to distinguish between these sequences based on the actual notes in the auditory display since GC or AT rich codons were not explicitly mapped to distinct pitch ranges (additionally recall that note selection is degenerate according to the genetic code).

**Fig. 2:**
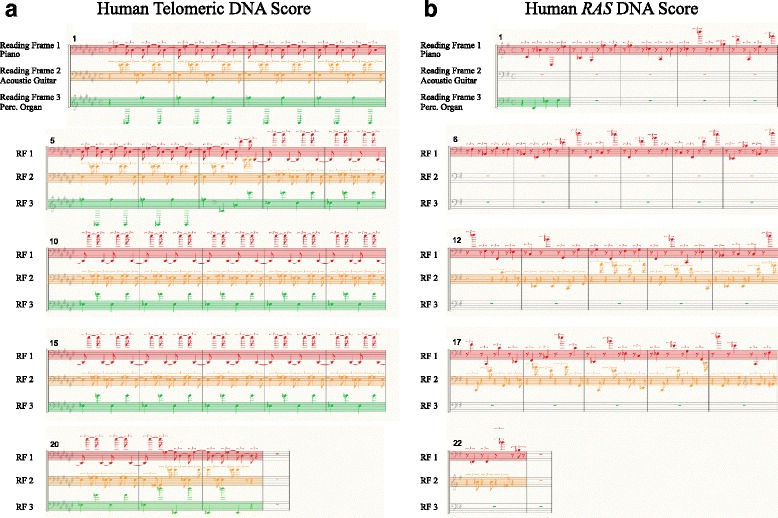
Musical scores representing the auditory displays of a) the Human Telomeric DNA sequence and b) the *RAS* DNA sequence. In each case, DNA sequences were sonified (using the ‘reading frames codons’ algorithm) to produce three staves of music that are played simultaneously. Each stave is labelled to show the reading frame it represents and the instrument used for its sonification. Measures are indicated at the top left of each stave

The input ‘Human Telomeric DNA’ sequence (see Table 3, Sequence 8) consists of a tandem array of the naturally occurring hexanucleotide repeat sequence TTAGGG [26] and contains insertion and substitution mutations. This was sonified with the ‘Reading frame codons’ algorithm and the ‘Ignore Start/Stop’ option to produces approximately 44 s of audio (Additional file 10) that has a melodic repetition that can be quickly and easily identified. Natural variation within Human Telomeric DNA sequences such as point mutations and insertions/deletions are common which are difficult to perceive by eye, however, these are easily perceived in the auditory display. The first section of this display is a repetitive six-note (two triplets) pattern indicative of an invariant hexanucleotide repeat. This is interrupted at 13 s by a brief flurry of notes giving rise to an alternative repetitive pattern. This frame shift mutation is also apparent in the musical score of the auditory display shown in Fig. 2a whereby during measure 7 the repetitive note pattern in all frames changes to a different repetitive pattern beginning in measure 8. Through sonification alone it is clear that there has been an insertion or deletion causing a frame shift since afterwards the repetitive phrase is mapped to different notes. A simple point mutation would only have caused a transient audio change without a change to the subsequent repetitive six-note pattern (as demonstrated by sonification of Sequence 2). These ‘Morse code blips’ highlights a change in the repetitive sequence and further visually inspection reveals the replacement of T with AG in the DNA sequence at bp 79 (as indicated by red text in Table 3). This clearly indicates the usefulness of the auditory display as an adjunct to visual inspection. Another distinct change can be heard at 40 s in the auditory display and visual inspection of the sequence reveals that this is due to the insertion of TG into the sequence at bp 241. These changes are not obvious by visual inspection of the sequence alone. The last 4 s of audio sound is identical to the start of the audio indicating that the sequence is again in the original reading frame. Overall three bases were inserted across the two mutation points and hence the notes have been assigned back to the same instrumentation.

The second repetitive DNA sequence to be analysed was the Human Alphoid repetitive DNA (see Table 3, Sequence 9), which consists of tandem arrays of the pentanucleotide sequence CCATT. With the ‘Start/Stop codons’ option set to ‘Ignore Start/Stop’, the first 16 s is derived from a synthetic alphoid sequence with no sequence variation. Again the audio from this sequence (Additional file 11) is clearly repetitive but paradoxically more complex than the previous hexanucleotide telomeric DNA sequence. This is because even though the sequence repeats after 5 base, three repeats of the entire sequence are required before the in-frame reoccurrence, i.e. the pattern is repeated every 5 codons or 15 bp. After the first 16 s, natural sequence variations occur and whilst these briefly interrupt the repetitive audio they are quickly passed and the pattern remains discernable. This is because the first mutations are point mutations, not insertions or deletions. The auditory display is more complex during the latter section and whist the repetitive nature of the audio is still evident no further conclusion can be drawn. Applying the default settings, i.e. the ‘Start/Stop codons’ option was reset to ‘Restart on ATG’ (as represented by Additional file 12), reveals the absence of any ATG codons and that two stop codons occur in alternate frames since only one instrument plays during the end section of the display.

### Sonification of complex DNA sequences

Sonification of the first exon of the human *RAS* gene [27] (see Table 3, Sequence 10, Additional file 13) with the ‘Reading frame codons’ algorithm and the ‘Silent until ATG’ option produces a distinctive auditory display. Clearly this sequence begins with ATG (the first codon of Reading frame 1), hence the piano instrument plays throughout the entire auditory display which is indicative of an open reading frame, often separated by two rest beats. Re-sonification of the sequence with the ‘Restart on ATG’ option selected (where all frames are audible at the start) highlights that the first codon in the second reading frame is a stop codon since the second instrument is silent, this can be observed in the score of the human *RAS* auditory display shown in Fig. 2b. Furthermore, reading frame 2 becomes audible again during the 12th measure since it is triggered by the occurrence of a start codon. This score highlights the silencing effect of a stop codon in reading frame 3 prior to the second measure. This also demonstrates that, in the absence of other influences on the DNA sequence, there is a one in four chance of there being a stop codon following a start codon since the last two bases of A*TG* start are the first two bases of the *TG*A stop. In conjunction with other visual clues and DNA analyses tools, the auditory display is useful to recognition a potential open frame in either reading frame 1, 2 or 3. Additionally the human *RAS* gene sequence was used to exemplify option to sonify the occurrence of stop and start codons using percussive sounds within the auditory display (i.e. by setting the ‘Highlight STOP START Codons’ to ‘yes’). This approach highlights the occurrence of multiple stop codons (using high frequency cymbals) in frames 2 and 3 (Additional file 14). The occurrence of multiple stop codons is not explicitly evident in the prior auditory display since the first occurrence has silenced the audio stream of the frame in which they later occur. This indicates that direct sonification of these codons as percussive instruments is useful.

In stark comparison to the sonification of a coding sequence such as the *RAS* exon is the sonification of a ribosomal DNA sequences, such as the yeast 15S ribosomal RNA (see Table 3, Sequence 11). This sequence was sonified in a similar way to the human *RAS* gene sequence (with the ‘Restart on ATG’ default option), to give an auditory display (Additional file 15) that is highly distinctive from the audio derived from the prior ORF or repetitive sequences. Overall, the rRNA display is characterised by extended passages of silence across three instruments because stop codons occur in close proximity in all reading frames. This sequence was also sonified with the ‘Highlight STOP START Codons’ to ‘yes’ (Additional file 16) so that the occurrence of stop codons could be detected as high frequency cymbals during these silent passages. Indeed there are sections where only cymbals are audible. There are approximately 20 short sections of silence throughout the 4 min auditory display. The triplet characteristic of the audio is mostly absent and many sections exhibit a sparsity of notes, that include many rest beats and often single instrument derived from either reading frame. This is characteristic of other test RNA sequences (available on the homepage) that are typically scattered with stop codons in multiple reading frames often resulting in regions of silence in the auditory display.  All code for website has been packaged as a single file called sonification.zip (see Additional file 17).

## Conclusion

Sonification algorithms based on the central dogma of molecular biology can help identify potential reading frames in complex DNA sequences and can identify mutations in repetitive DNA sequences that are obscure by visual inspection alone. A particularly useful approach is to sonify codons in all three reading frames and to use start and stop codons as switches to turn an audio instrument on or off.

In the future it may be useful to sonify other important DNA motifs such as transcription factor binding sites, restriction endonucleases sites etc. to unique sounds to highlight their occurrence.

Whilst mapping three nucleotides to a note is instructive it may also be useful to map the output of more complex Bayesian methods of sequence analyses to an auditory display. Additionally it may be useful to use an auditory display to highlight variation in multiple sequence alignments or genomic variations such as single nucleotide polymorphisms. In summary, this study presents a proof-of-concept to show that properties of a DNA sequence can be identified through sonification and provides an impetus for the inclusion of auditory displays within the toolkit of DNA sequence browsers, as an adjunct to existing visual and analytical tools.

## Availability and requirements

**Project name:** DNA Sonification

**Project home page:** http://dnasonification.org

**Operating system:** Platform independent

**Programming language:** HTML, PHP and javascript

**Other requirements:** Web browser equipped with plugin to play MIDI file, e.g. the Jazz-Plugin

**License:** GNU GPL

**Any restrictions to use by non-academics:** None

## Additional files

Additional file 1: Auditory Display of G Sequence. An audio file produced by sonification of the sequence using the default ‘Reading frame codons’ algorithm, the ‘Start/Stop codons’ option was set to ‘Restart on ATG’ and the ‘Highlight STOP START Codons’ option was set to ‘no’. (MP3 625 kb)

Additional file 2: Auditory Display of Mutated G Sequence. An audio file produced by sonification of the sequence using the default ‘Reading frame codons’ algorithm, the ‘Start/Stop codons’ option was set to ‘Restart on ATG’ and the ‘Highlight STOP START Codons’ option was set to ‘no’. (MP3 573 kb)

Additional file 3: Auditory Display of G Sequence with STOP in the 1st Reading Frame. An audio file produced by sonification of the sequence using the default ‘Reading frame codons’ algorithm, the ‘Start/Stop codons’ option was set to ‘Restart on ATG’ and the ‘Highlight STOP START Codons’ option was set to ‘no’. (MP3 573 kb)

Additional file 4: Auditory Display of G Sequence with STOP in all Reading Frames, version A. An audio file produced by sonification of the sequence using the default ‘Reading frame codons’ algorithm, the ‘Start/Stop codons’ option was set to ‘Restart on ATG’ and the ‘Highlight STOP START Codons’ option was set to ‘no’. (MP3 573 kb)

Additional file 5: Auditory Display of G Sequence with STOP in all Reading Frames, version B. An audio file produced by sonification of the sequence using the default ‘Reading frame codons’ algorithm, the ‘Start/Stop codons’ option was set to ‘Restart after 10 codons’ and the ‘Highlight STOP START Codons’ option was set to ‘no’. (MP3 573 kb)

Additional file 6: Auditory Display of G Sequence with STOP in All, START in 1st RF. An audio file produced by sonification of the sequence using the default ‘Reading frame codons’ algorithm, the ‘Start/Stop codons’ option was set to ‘Restart on ATG’ and the ‘Highlight STOP START Codons’ option was set to ‘no’. (MP3 573 kb)

Additional file 7: Auditory Display of AT only DNA. An audio file produced by sonification of the sequence using the default ‘Reading frame codons’ algorithm, the ‘Start/Stop codons’ option was set to ‘Restart on ATG’ and the ‘Highlight STOP START Codons’ option was set to ‘no’. (MP3 1459 kb)

Additional file 8: Auditory Display of AT only DNA. An audio file produced by sonification of the sequence using the default ‘Reading frame codons’ algorithm, the ‘Start/Stop codons’ option was set to ‘Ignore Start/Stop’ and the ‘Highlight STOP START Codons’ option was set to ‘no’. (MP3 1459 kb)

Additional file 9: Auditory Display of GC only DNA. An audio file produced by sonification of the sequence using the default ‘Reading frame codons’ algorithm, the ‘Start/Stop codons’ option was set to ‘Restart on ATG’ and the ‘Highlight STOP START Codons’ option was set to ‘no’. (MP3 1459 kb)

Additional file 10: Auditory Display of Human Telomeric DNA. An audio file produced by sonification of the sequence using the default ‘Reading frame codons’ algorithm, the ‘Start/Stop codons’ option was set to ‘Ignore Start/Stop’ and the ‘Highlight STOP START Codons’ option was set to ‘no’. (MP3 1479 kb)

Additional file 11: Auditory Display of Alphoid Repetitive DNA. An audio file produced by sonification of the sequence using the default ‘Reading frame codons’ algorithm, the ‘Start/Stop codons’ option was set to ‘Ignore Start/Stop’ and the ‘Highlight STOP START Codons’ option was set to ‘no’. (MP3 2089 kb)

Additional file 12: Auditory Display of Alphoid Repetitive DNA. An audio file produced by sonification of the sequence using the default ‘Reading frame codons’ algorithm, the ‘Start/Stop codons’ option was set to ‘Restart on ATG’ and the ‘Highlight STOP START Codons’ option was set to ‘no’. (MP3 2089 kb)

Additional file 13: Auditory Display of *RAS* coding sequence (cDNA). An audio file produced by sonification of the sequence using the default ‘Reading frame codons’ algorithm, the ‘Start/Stop codons’ option was set to ‘Silent until ATG’ and the ‘Highlight STOP START Codons’ option was set to ‘no’. (MP3 3094 kb)

Additional file 14: Auditory Display of *RAS* coding sequence (cDNA). An audio file produced by sonification of the sequence using the default ‘Reading frame codons’ algorithm, the ‘Start/Stop codons’ option was set to ‘Restart on ATG’ and the ‘Highlight STOP START Codons’ option was set to ‘yes. (MP3 3094 kb)

Additional file 15: Auditory Display of 15S rRNA non-coding RNA. An audio file produced by sonification of the sequence using the default ‘Reading frame codons’ algorithm, the ‘Start/Stop codons’ option was set to ‘Restart on ATG’ and the ‘Highlight STOP START Codons’ option was set to ‘no’. (MP3 8714 kb)

Additional file 16: Auditory Display of 15S rRNA non-coding RNA. An audio file produced by sonification of the sequence using the default ‘Reading frame codons’ algorithm, the ‘Start/Stop codons’ option was set to ‘Restart on ATG’ and the ‘Highlight STOP START Codons’ option was set to ‘yes. (MP3 8714 kb)

Additional file 17: Code for website; including html, php and associated files. (ZIP 49453 kb)

## Abbreviations

MIDI: Musical Instrument Digital Interface
