# Supplementary material for: An auditory display tool for DNA sequence analysis
Source: BMC Bioinformatics. 2017 Apr 24;18:221. doi: 10.1186/s12859-017-1632-x (PMC5404335; doi:10.1186/s12859-017-1632-x)
Supplement: Supplementary file 17 — Code for website; including html, php and associated files. (ZIP 49453 kb) [file 12859_2017_1632_MOESM17_ESM.zip › sonification/GeneralMidi.html]

General MIDI Sounds


This page requires Jazz-Plugin ...

Piano

0 Acoustic Grand Piano

1 Bright Acoustic Piano

2 Electric Grand Piano

3 Honky-tonk Piano

4 Electric Piano 1

5 Electric Piano 2

6 Harpsichord

7 Clavinet

Chromatic Percussion

8 Celesta

9 Glockenspiel

10 Music Box

11 Vibraphone

12 Marimba

13 Xylophone

14 Tubular Bells

15 Dulcimer

Organ

16 Drawbar Organ

17 Percussive Organ

18 Rock Organ

19 Church Organ

20 Reed Organ

21 Accordion

22 Harmonica

23 Tango Accordion

Guitar

24 Acoustic Guitar (nylon)

25 Acoustic Guitar (steel)

26 Electric Guitar (jazz)

27 Electric Guitar (clean)

28 Electric Guitar (muted)

29 Overdriven Guitar

30 Distortion Guitar

31 Guitar Harmonics

Bass

32 Acoustic Bass

33 Electric Bass (finger)

34 Electric Bass (pick)

35 Fretless Bass

36 Slap Bass 1

37 Slap Bass 2

38 Synth Bass 1

39 Synth Bass 2

Strings

40 Violin

41 Viola

42 Cello

43 Contrabass

44 Tremolo Strings

45 Pizzicato Strings

46 Orchestral Harp

47 Timpani

Ensemble

48 String Ensemble 1

49 String Ensemble 2

50 Synth Strings 1

51 Synth Strings 2

52 Choir Aahs

53 Voice Oohs

54 Synth Choir

55 Orchestra Hit

Brass

56 Trumpet

57 Trombone

58 Tuba

59 Muted Trumpet

60 French Horn

61 Brass Section

62 Synth Brass 1

63 Synth Brass 2

Reed

64 Soprano Sax

65 Alto Sax

66 Tenor Sax

67 Baritone Sax

68 Oboe

69 English Horn

70 Bassoon

71 Clarinet

Pipe

72 Piccolo

73 Flute

74 Recorder

75 Pan Flute

76 Blown Bottle

77 Shakuhachi

78 Whistle

79 Ocarina

Synth Lead

80 Lead 1 (square)

81 Lead 2 (sawtooth)

82 Lead 3 (calliope)

83 Lead 4 (chiff)

84 Lead 5 (charang)

85 Lead 6 (voice)

86 Lead 7 (fifths)

87 Lead 8 (bass + lead)

Synth Pad

88 Pad 1 (new age)

89 Pad 2 (warm)

90 Pad 3 (polysynth)

91 Pad 4 (choir)

92 Pad 5 (bowed)

93 Pad 6 (metallic)

94 Pad 7 (halo)

95 Pad 8 (sweep)

Synth Effects

96 FX 1 (rain)

97 FX 2 (soundtrack)

98 FX 3 (crystal)

99 FX 4 (atmosphere)

100 FX 5 (brightness)

101 FX 6 (goblins)

102 FX 7 (echoes)

103 FX 8 (sci-fi)

Ethnic

104 Sitar

105 Banjo

106 Shamisen

107 Koto

108 Kalimba

109 Bagpipe

110 Fiddle

111 Shanai

Percussive

112 Tinkle Bell

113 Agogo

114 Steel Drums

115 Woodblock

116 Taiko Drum

117 Melodic Tom

118 Synth Drum

119 Reverse Cymbal

Sound Effects

120 Guitar Fret Noise

121 Breath Noise

122 Seashore

123 Bird Tweet

124 Telephone Ring

125 Helicopter

126 Applause

127 Gunshot
