# Supplementary material for: An auditory display tool for DNA sequence analysis
Source: BMC Bioinformatics. 2017 Apr 24;18:221. doi: 10.1186/s12859-017-1632-x (PMC5404335; doi:10.1186/s12859-017-1632-x)
Supplement: Supplementary file 17 — Code for website; including html, php and associated files. (ZIP 49453 kb) [file 12859_2017_1632_MOESM17_ESM.zip › sonification/JZZ-modules-master/html/JZZ_HelloMIDI.html]

Hello MIDI!


Home »
Examples »
Write MIDI File

# Write MIDI File

This page requires Jazz-Plugin ...

## How it works

Save the above link as *\*.kar* file and open it in MIDI/karaoke player.

If the approppriate plugin (most likely, *QuickTime*) is enabled in your browser,
you will also see an embedded object next to the link.

The code below creates MIDI file from scratch and writes it into HTML document.

(C) 2011-16 Jazz-Soft
