# Supplementary material for: An auditory display tool for DNA sequence analysis
Source: BMC Bioinformatics. 2017 Apr 24;18:221. doi: 10.1186/s12859-017-1632-x (PMC5404335; doi:10.1186/s12859-017-1632-x)
Supplement: Supplementary file 17 — Code for website; including html, php and associated files. (ZIP 49453 kb) [file 12859_2017_1632_MOESM17_ESM.zip › sonification/JZZ-modules-master/html/JZZ_PlayMidiFile.html]

Play MIDI File


This page requires
Jazz-Plugin ...

Play
Pause
Stop

*The Sonified audio playback requires Jazz-Plugin - best in Safari*

PHP
$bas64MIDfile = (base64\_encode(file\_get\_contents("\_HumanTelomericDNA.mid" )));
echo "$bas64MIDfile";
echo "foo bar";
?
