# Supplementary material for: An auditory display tool for DNA sequence analysis
Source: BMC Bioinformatics. 2017 Apr 24;18:221. doi: 10.1186/s12859-017-1632-x (PMC5404335; doi:10.1186/s12859-017-1632-x)
Supplement: Supplementary file 17 — Code for website; including html, php and associated files. (ZIP 49453 kb) [file 12859_2017_1632_MOESM17_ESM.zip › sonification/JZZ-modules-master/html/midijs.html]

JZZ.synth.MIDIjs


# JZZ.synth.MIDIjs

```
JZZ.synth.MIDIjs('MIDI.js', {...})

  .note(0, 'C5', 127, 500).wait(500)
  .note(0, 'E5', 127, 500).wait(500)
  .note(0, 'G5', 127, 500).wait(500)
  .note(0, 'C6', 127, 500);
```


```
JZZ.synth.MIDIjs.register('MIDI.js', {...});
JZZ().openMidiOut('MIDI.js')
  .note(0, 'C5', 127, 500).wait(500)
  .note(0, 'E5', 127, 500).wait(500)
  .note(0, 'G5', 127, 500).wait(500)
  .note(0, 'C6', 127, 500);
```

NOTE: MIDI.js may not work in all browsers if called from the local file.
