# Supplementary material for: An auditory display tool for DNA sequence analysis
Source: BMC Bioinformatics. 2017 Apr 24;18:221. doi: 10.1186/s12859-017-1632-x (PMC5404335; doi:10.1186/s12859-017-1632-x)
Supplement: Supplementary file 17 — Code for website; including html, php and associated files. (ZIP 49453 kb) [file 12859_2017_1632_MOESM17_ESM.zip › sonification/JZZ-modules-master/html/timbre.html]

JZZ.synth.Timbre


# JZZ.synth.Timbre

```
JZZ.synth.Timbre.register('Timbre', synth3)
JZZ().openMidiOut('Timbre')
  .note(0, 'C5', 127, 500).wait(500)
  .note(0, 'E5', 127, 500).wait(500)
  .note(0, 'G5', 127, 500).wait(500)
  .note(0, 'C6', 127, 500);
}
```


```
JZZ.synth.Timbre('Timbre', synth)

  .note(0, 'C5', 127, 500).wait(500)
  .note(0, 'E5', 127, 500).wait(500)
  .note(0, 'G5', 127, 500).wait(500)
  .note(0, 'C6', 127, 500);
}
```

NOTE: Timbre.js may not work in IE9 and some other browsers.
