# Supplementary material for: An auditory display tool for DNA sequence analysis
Source: BMC Bioinformatics. 2017 Apr 24;18:221. doi: 10.1186/s12859-017-1632-x (PMC5404335; doi:10.1186/s12859-017-1632-x)
Supplement: Supplementary file 17 — Code for website; including html, php and associated files. (ZIP 49453 kb) [file 12859_2017_1632_MOESM17_ESM.zip › sonification/JZZ-modules-master/html/XXXindex.html]

JZZ-modules


# JZZ-modules

JZZ.synth.MIDIjs.js
- connect MIDI.js to JZZ.js

JZZ.synth.Timbre.js
- wrapper for a Timbre.js synth

JZZ.synth.OSC.js
- Web Audio oscillator-based synth

JZZ.input.Querty.js
- wrapper for a Qwerty Hancock piano.

JZZ.input.Kbd.js
- HTML/JavaScript piano.

JZZ.input.Knobs.js
- Sliders and Knobs.

JZZ.input.ASCII.js
- ASCII keyboard as MIDI input.
