# Supplementary material for: An auditory display tool for DNA sequence analysis
Source: BMC Bioinformatics. 2017 Apr 24;18:221. doi: 10.1186/s12859-017-1632-x (PMC5404335; doi:10.1186/s12859-017-1632-x)
Supplement: Supplementary file 17 — Code for website; including html, php and associated files. (ZIP 49453 kb) [file 12859_2017_1632_MOESM17_ESM.zip › sonification/JZZ-modules-master/javascript/JZZ_ReadMIDI.html]

Read MIDI File


# Read MIDI File

## from file:

## from URL:

## from string: Read...

Files accepted: SMF (\*.mid, \*.kar), RMID (\*.rmi)
