# Supplementary material for: An auditory display tool for DNA sequence analysis
Source: BMC Bioinformatics. 2017 Apr 24;18:221. doi: 10.1186/s12859-017-1632-x (PMC5404335; doi:10.1186/s12859-017-1632-x)
Supplement: Supplementary file 17 — Code for website; including html, php and associated files. (ZIP 49453 kb) [file 12859_2017_1632_MOESM17_ESM.zip › sonification/midi_class_v175/documentation.htm]

PHP MIDI CLASS


# PHP MIDI CLASS

## METHOD OVERVIEW

addMsg()  
deleteMsg()  
deleteTrack()  
downloadMidFile()  
getBpm()  
getDrumkitList()  
getDrumset()  
getInstrumentList()  
getMid()  
getMsg()  
getMsgCount()  
getNoteList()  
getTempo()  
getTimebase()  
getTrack()  
getTrackCount()  
getTrackTxt()  
getTxt()  
getXml()  
importMid()  
importTrackTxt()  
importTxt()  
importXml()  
insertMsg()  
newTrack()  
open()  
playMidFile()  
saveMidFile()  
setBpm()  
setTempo()  
setTimebase()  
soloTrack()  
transpose()  
transposeTrack()  
  
**RTTL Class (entension/child class)**  
getRttl()  
importRttl()  

## METHODS IN DETAIL

### Binary Input

importMid($smf\_path [, $tn])  
imports standard MIDI file (SMF, \*.mid) or RMID files (\*.rmi) of type 0 or 1. If optional 2. parameter $tn is set, only track number $tn is imported (similar effect as soloTrack(), but much faster, as only one track is parsed).
  
  
Example:  
$midi->importMid('music/song1.mid');

### Text Input

importTxt($txt)  
imports MIDI song in MF2T/T2MF ASCII format (e.g. fetched from database). If a line "TimestampType=Delta" is found at the beginning of a track (next line after "MTrk"), all timestamps of this track are interpreted as relative (delta) values, otherwise as absolute values.
  
  
Notice:  
Relative timestamps and the "TimestampType=Delta" are not compatible with the original MF2T/T2MF format!
  
  
Example:  
$midi->importTxt($txt);
  
  
  
importTrackTxt($txt [, $tn])  
imports MIDI track in MF2T/T2MF format. If optional track number $tn is specified, track $tn is replaced, otherwise new track is appended. If a line "TimestampType=Delta" is found at the beginning of the track (next line after "MTrk"), all timestamps of this track are interpreted as relative (delta) values, otherwise as absolute values.
  
  
Notice:  
Relative timestamps and the "TimestampType=Delta" are not compatible with the original MF2T/T2MF format!
  
  
Example:  
$midi->importTrackTxt($txt, 2);

### XML Input

importXML($txt)  
imports MIDI song in MIDI XML format
  
  
Example:  
$midi->importXML($xmlStr);

### Binary Output

getMid()  
returns MIDI song as binary MIDI string (SMF).
  
  
Example:  
$smf = $midi->getMid();
  
  
  
saveMidFile($mid\_path)  
saves MIDI song as standard MIDI file (SMF).
  
  
Example:  
$midi->saveMidFile('/tmp/song1.mid');
  
  
  
playMidFile($file [,$visible=1,$autostart=1,$loop=1,$plug=''])  
embeds MIDI file in web page. Optional parameters for visibility, autostart and looping (1=yes,0=no). Optional parameter $plug determines preferred plugin, it can be 'bk' (beatnik),
'qt' (QuickTime) or 'wm' (Windows Media Player), if it's omitted or empty string (''), the default player for MIDI files is used (<embed type="audio/midi"...>).
  
  
Examples:  
$midi->playMidFile($file);  
$midi->playMidFile($file,0,1,0,'bk');
  
  
  
downloadMidFile($output [,$file])  
if parameter $file is specified, starts download of file $file, otherwise the current midi song is downloaded from memory. $output is the proposed filename for saving.
  
  
Example:  
$midi->downloadMidFile('song1.mid', '/tmp/~tmp1.mid');

### Text Output

getTxt([$ttype=0])  
returns MIDI song in MF2T/T2MF format (e.g. to save to database). Optional Parameter $ttype determines timestamp type, if $ttype==0 (or omitted), the timestamps are absolute values and the result is compatible to the original MF2T/T2MF format, if $ttype==1, timestamps are relative values (not compatible to original MF2T/T2MF format):
  
  
Notice:  
As messages are internally stored with absolute timestamps, getTxt() is much faster than getTxt(1);
  
  
Example:  
$str = $midi->getTxt();
  
  
  
getTrackTxt($tn[,$ttype=0])  
returns track number $tn in MF2T/T2MF format. For optional parameter $ttype see getTxt().
  
  
Example:  
$str = $midi->getTrackTxt(2,1);

### XML Output

getXml([$ttype=0])  
returns MIDI song as MIDI XML. Optional Parameter $ttype determines timestamp type, if $ttype==0 (or omitted), the timestamps are absolute values, if $ttype==1 timestamps are relative (delta) values. The output is valid XML according to the MIDI XML standard v0.9 by MusicXML/Recordare.
  
  
Notice:  
As messages are internally stored with absolute timestamps, getXml() is much faster than getXml(1);
  
  
Example:  
$xmlStr = $midi->getXml();

### Info

getTempo()  
returns MIDI tempo value (0 if not set).
  
  
  
getBpm()  
returns tempo as beats per minute (0 if tempo not set).
  
  
  
getTimebase()  
returns timebase value.
  
  
  
getTrackCount()  
returns number of tracks.
  
  
  
getTrack($tn)  
returns track number $tn as array of msg strings in MF2T/T2MF format.
  
  
Example:  
$track = $midi->getTrack(2);
  
  
  
getMsgCount()  
returns number of messages of track number $tn.
  
  
Example:  
$msgCnt = $midi->getMsgCount(2);
  
  
  
getMsg($tn,$mn)  
returns message string number $mn of track number $tn. The message string is in MF2T/T2MF format.
  
  
Example:  
$msgStr = $midi->getMsg(2, 23);

### Generation/Manipulation

open([$timebase=480])  
creates(resets) new empty MIDI song for further processing (eg. with newTrack() and addMsg()). Optional parameter $timebase to set its timebase (default = 480).
  
  
Example:  
$midi->open(240);
  
  
  
newTrack()  
adds new empty track, returns its track number.
  
  
Example:  
$newTrck = $midi->newTrack();
  
  
  
addMsg($tn, $msgStr[, $ttype=0])  
appends message string $msgStr to end of track $tn. If $ttype==0 or omitted, timestamp is interpreted as absolute value, if $ttype==1, it's interpreted as relative (delta) value. The message string has to be in MF2T/T2MF format.
  
  
Notice:  
addMsg() is faster then insertMsg(), but it does not check for correct position according to time.
  
  
Example:  
$midi->addMsg(1, "4800 On ch=1 n=66 v=80");
  
  
  
insertMsg($tn,$msgStr)  
adds message at adequate position of track number $tn (slower than addMsg).  
If other messages with same time are found, new message is inserted before
those. The message string has to be in MF2T/T2MF format.
  
  
Example:  
$midi->insertMsg(0, "0 Meta Text \"created by fluxus\"");
  
  
  
deleteMsg($tn,$mn)  
deletes message $mn of track $tn.
  
  
  
setBpm($bpm)  
sets tempo corresponding to given beats per minute $bpm.
  
  
  
setTempo($tempo)  
sets tempo by replacing set tempo msg in track 0 (or adding new track 0).
  
  
  
setTimebase($tb)  
sets timebase
  
  
  
deleteTrack($tn)  
deletes track number $tn.
  
  
  
soloTrack($tn)  
deletes all tracks except track number $tn (and $track 0 which contains tempo info).
  
  
  
transpose($dn)  
transposes song by $dn (positive or negative) half tone steps.
  
  
Example:  
$midi->transpose(12); // 1 octave up
  
  
  
transposeTrack($tn, $dn)  
transposes all notes of track number $tn by $dn (positive or negative) half tone steps.
  
  
Example:  
$midi->transposeTrack(1, -12); // 1 octave down

### Utilities

getDrumkitList()  
returns list of standard gm drum kit names (associative array, key=number, value=name).
  
  
  
getDrumset()  
returns list of gm drumset instrument names (associative array, key=number, value=name).
  
  
  
getInstrumentList()  
returns list of standard instrument names.
  
  
  
getNoteList()  
returns list of note names ('C0',...).

### RTTL Extension

getRttl([$title='', $tn=-1]])  
returns RTTL ringtone as string. If optional parameter $title is specified, this will be the RTTL name (max. 10 characters), if it's "" or omitted, the method looks for a TrkName meta event, and if there is none, name will be "mid2rttl". The optional parameter $tn determines the tracknumber that will be used, if it's <0 or omitted, the first track that contains note events will be used.
  
  
Example:  
$rttlStr = $rttl->getRttl();
  
  
  
importRttl($rttlStr)  
imports a RTTL ringtone as string (creates a new midi song of type 0).
  
  
Example:  
$rttl->importRttl($rttlStr);
  
  

## MF2T/T2MF text format

excerpt of readme.txt in mf2t.zip

```
Format of the textfile:
-----------------------

File header:        Mfile <format> <ntrks> <division>
Start of track:     MTrk
End of track:       TrkEnd

Note On:            On <ch> <note> <vol>
Note Off:           Off <ch> <note> <vol>
Poly Pressure:      PoPr[PolyPr] <ch> <note> <val>
Channel Pressure:   ChPr[ChanPr] <ch> <val>
Controller
parameter:          Par[Param] <ch> <con> <val>
Pitch bend:         Pb <ch> <val>
Program change:     PrCh[ProgCh] <ch> <prog>
Sysex message:      SysEx <hex>


Sequence nr:        Seqnr <num>
Key signature:      KeySig <num> <manor>
Tempo:              Tempo <num>
Time signature:     TimeSig <num>/<num> <num> <num>
SMPTE event:        SMPTE <num> <num> <num> <num> <num>

Meta text events:   Meta <texttype> <string>
Meta end of track:  Meta TrkEnd
Sequencer specific: SeqSpec <type> <hex>
Misc meta events:   Meta <type> <hex>

The <> have the following meaning:

<ch>        ch=<num>
<note>      n=<noteval>  [note=<noteval>]
<vol>       v=<num> [vol=<num>]
<val>       v=<num> [val=<num>]
<con>       c=<num> [con=<num>]
<prog>      p=<num> [prog=<num>]
<manor>     minor or major
<noteval>   either a <num> or A-G optionally followed by #,
            followed by <num> without intermediate spaces.

<texttype>  Text Copyright SeqName TrkName InstrName Lyric Marker Cue or <type>
<type>      a hex number of the form 0xab
<hex>       a sequence of 2-digit hex numbers (without 0x) separated by space
<string>    a string between double quotes (like "text").

Channel numbers are 1-based, all other numbers are as they appear in the midifile.

<division>  is either a positive number (giving the time resolution in
            clicks per quarter note) or a negative number followed by a positive
            number (giving SMPTE timing).

<format> <ntrks> <num> are decimal numbers.

The <num> in the Pb is the real value (two midibytes combined).
In Tempo it is a long (32 bits) value. Others are in the interval 0-127.
The SysEx sequence contains the leading F0 and the trailing F7.
```

## MIDI XML format

(for details see Standard MIDI File DTD by Recordare)
  
  
**Supported elements:**

```
<MIDIFile>...</MIDIFile>
<Format>...</Format>
<TrackCount>...</TrackCount>
<TicksPerBeat>...</TicksPerBeat>
<TimestampType>...</TimestampType>
```

**Supported events:**

```
<ProgramChange Channel="..." Number="..."/>
<NoteOn Channel="..." Note="..." Velocity="..."/>
<NoteOff Channel="..." Note="..." Velocity="..."/>
<PolyKeyPressure Channel="..." Note="..." Pressure="..."/>
<ControlChange Channel="..." Control="..." Value="..."/>
<ChannelKeyPressure Channel="..." Pressure="..."/>
<PitchBendChange Channel="..." Value="..."/>
<SequenceNumber Value="..."/>
<TextEvent>...</TextEvent>
<CopyrightNotice>...</CopyrightNotice>
<TrackName>...</TrackName>
<InstrumentName>...</InstrumentName>
<Lyric>...</Lyric>
<Marker>...</Marker>
<CuePoint>...</CuePoint>
<EndOfTrack/>
<MIDIChannelPrefix Value="..."/>
<SetTempo Value="..."/>
<SMPTEOffset TimeCodeType="1" Hour="..." Minute="..." Second="..." Frame="..." FractionalFrame="..."></SMPTEOffset>
<TimeSignature Numerator="..." LogDenominator="..." MIDIClocksPerMetronomeClick="..." ThirtySecondsPer24Clocks="..."/>
<KeySignature Fifths="..." Mode="..."/>
<SequencerSpecific>...</SequencerSpecific>
<SystemExclusive>...</SystemExclusive>
```

**Not (yet) supported elements:**

```
<!-- for SMPTE time codes -->
<FrameRate>...</FrameRate>
<TicksPerFrame>...<TicksPerFrame>
```

**Not (yet) supported events:**

```
<EndOfExclusive>...</EndOfExclusive>
<ProgramName>...</ProgramName>
<DeviceName>...</DeviceName>
<XMFPatchTypePrefix Value="..."/>
<Port Value="..."/>
<OtherMetaEvent Number="...">...</OtherMetaEvent>
```

  
  
  
  
  
  
