# Supplementary material for: An auditory display tool for DNA sequence analysis
Source: BMC Bioinformatics. 2017 Apr 24;18:221. doi: 10.1186/s12859-017-1632-x (PMC5404335; doi:10.1186/s12859-017-1632-x)
Supplement: Supplementary file 17 — Code for website; including html, php and associated files. (ZIP 49453 kb) [file 12859_2017_1632_MOESM17_ESM.zip › sonification/midi_class_v175/midifile.htm]

MIDI File Format


# The MIDI File Format

The *Standard MIDI File* (SMF) is a file format specifically designed to store the data
that a sequencer records and plays (whether that sequencer be software or hardware based).

This format stores the standard MIDI messages (ie, status bytes with appropriate data bytes)
plus a time-stamp for each message (ie, a series of bytes that represent how many clock pulses
to wait before "playing" the event). The format allows saving information about tempo, pulses
per quarter note resolution (or resolution expressed in divisions per second, ie SMPTE setting),
time and key signatures, and names of tracks and patterns. It can store multiple patterns and
tracks so that any application can preserve these structures when loading the file.

**NOTE:** A *track* usually is analogous to one musical part, such as a Trumpet part. A
*pattern* would be analogous to all of the musical parts (ie, Trumpet, Drums, Piano, etc)
for a song, or excerpt of a song.

The format was designed to be generic so that any sequencer could read or write such a file
without losing the most important data, and flexible enough for a particular application to store its
own proprietary, "extra" data in such a way that another application won't be confused when
loading the file and can safely ignore this extra stuff that it doesn't need. Think of the MIDI file
format as a musical version of an ASCII text file (except that the MIDI file contains binary data
too), and the various sequencer programs as text editors all capable of reading that file. But,
unlike ASCII, MIDI file format saves data in *chunks* (ie, groups of bytes preceded by an
ID and size) which can be parsed, loaded, skipped, etc. Therefore, it can be easily extended to
include a program's proprietary info. For example, maybe a program wants to save a "flag byte"
that indicates whether the user has turned on an audible metronome click. The program can put
this flag byte into a MIDI file in such a way that another application can skip this byte without
having to understand what that byte is for. In the future, the MIDI file format can also be
extended to include new "official" chunks that all sequencer programs may elect to load and use.
This can be done without making old data files obsolete (ie, the format is designed to be
extensible in a backwardly compatible way).

## What's a Chunk?

Data is always saved within a *chunk*. There can be many chunks inside of a MIDI
file. Each chunk can be a different size (ie, where size refers to how many bytes are contained
in the chunk). The data bytes in a chunk are related in some way. A chunk is simply a group of
related bytes.

Each chunk begins with a 4 character (ie, 4 ascii bytes) *ID* which tells what "type"
of chunk this is. The next 4 bytes (all bytes are comprised of 8 bits) form a 32-bit length (ie,
size) of the chunk. **All chunks must begin with these two fields** (ie, 8 bytes), which
are referred to as the *chunk header*.

**NOTE:** The *Length* does not include the 8 byte chunk header. It simply tells you how many
bytes of data are in the chunk *following this header*.

Here's an example header (with bytes expressed in hex):

4D 54 68 64 00 00 00 06

Note that the first 4 bytes make up the ascii ID of **MThd** (ie, the first four
bytes are the ascii values for 'M', 'T', 'h', and 'd'). The next 4 bytes tell us that there should be
6 more data bytes in the chunk (and after that we should find the next chunk header or the end of
the file).

In fact, all MIDI files begin with this *MThd header* (and that's how you know that it's
a MIDI file).

**NOTE:** The 4 bytes that make up the *Length* are stored in Motorola 68000 byte order, not
Intel reverse byte order (ie, the 06 is the fourth byte instead of the first of the four). All multiple
byte fields in a MIDI file follow this standard, often called "Big Endian" form.

## MThd Chunk

The MThd header has an ID of **MThd**, and a Length of **6**.

Let's examine the 6 data bytes (which follow the above, 8 byte header) in an MThd chunk.

The first two data bytes tell the *Format* (which I prefer to call *Type*).
There are actually 3 different types (ie, formats) of MIDI files. A type of 0 means that the file
contains one single track containing midi data on possibly all 16 midi channels. If your sequencer
sorts/stores all of its midi data in one single block of memory with the data in the order that it's
"played", then it should read/write this type. A type of 1 means that the file contains one or
more simultaneous (ie, all start from an assumed time of 0) tracks, perhaps each on a single midi
channel. Together, all of these tracks are considered one sequence or pattern. If your
sequencer separates its midi data (i.e. tracks) into different blocks of memory but plays them back
simultaneously (ie, as one "pattern"), it will read/write this type. A type of 2 means that the
file contains one or more sequentially independant single-track patterns. f your sequencer
separates its midi data into different blocks of memory, but plays only one block at a time (ie,
each block is considered a different "excerpt" or "song"), then it will read/write this type.

The next 2 bytes tell how many tracks are stored in the file, *NumTracks*. Of course,
for format type 0, this is always 1. For the other 2 types, there can be numerous tracks.

The last two bytes indicate how many Pulses (i.e. clocks) Per Quarter Note (abbreviated as PPQN) resolution the
time-stamps are based upon, *Division*. For example, if your sequencer has 96 ppqn,
this field would be (in hex):

00 60

Alternately, if the first byte of Division is negative, then this represents the division
of a second that the time-stamps are based upon. The first byte will be -24, -25, -29, or -30,
corresponding to the 4 SMPTE standards representing frames per second. The second byte (a
positive number) is the resolution within a frame (ie, subframe). Typical values may be 4 (MIDI
Time Code), 8, 10, 80 (SMPTE bit resolution), or 100.

You can specify millisecond-based timing by the data bytes of -25 and 40 subframes.

Here's an example of a complete MThd chunk (with header):

```
4D 54 68 64     MThd ID
00 00 00 06     Length of the MThd chunk is always 6.
00 01           The Format type is 1.
00 02           There are 2 MTrk chunks in this file.
E7 28           Each increment of delta-time represents a millisecond.
```

## MTrk Chunk

After the MThd chunk, you should find an *MTrk chunk*, as this is the only other
currently defined chunk. (If you find some other chunk ID, it must be proprietary to some other
program, so skip it by ignoring the following data bytes indicated by the chunk's
Length).

An MTrk chunk contains all of the midi data (with timing bytes), plus optional non-midi data
for **one track**. Obviously, you should encounter as many MTrk chunks in the file as the
MThd chunk's NumTracks field indicated.

The MTrk header begins with the ID of *MTrk*, followed by the Length
(ie, number of data bytes to read for this track). The Length will likely be different for
each track. (After all, a track containing the violin part for a Bach concerto will likely contain
more data than a track containing a simple 2 bar drum beat).

## Variable Length Quantities -- Event's Time

Think of a track in the MIDI file in the same way that you normally think of a track in a
sequencer. A sequencer track contains a series of *events*. For example, the first
event in the track may be to sound a middle C note. The second event may be to sound the
E above middle C. These two events may both happen at the same time. The third event may
be to release the middle C note. This event may happen a few musical beats after the first
two events (ie, the middle C note is held down for a few musical beats). Each event has a
"time" when it must occur, and the events are arranged within a "chunk" of memory in the order
that they occur.

In a MIDI file, an event's "time" precedes the data bytes that make up that event itself. In
other words, the bytes that make up the event's time-stamp come first. A given event's
time-stamp is referenced from the previous event. For example, if the first event occurs 4 clocks
after the start of play, then its "delta-time" is 04. If the next event occurs simultaneously with
that first event, its time is 00. So, a delta-time is the duration (in clocks) between an event
and the preceding event.

**NOTE:** Since all tracks start with an assumed time of 0, the first event's delta-time is referenced
from 0.

A delta-time is stored as a series of bytes which is called a *variable length
quantity*. Only the first 7 bits of each byte is significant (right-justified; sort of like an
ASCII byte). So, if you have a 32-bit delta-time, you have to unpack it into a series of 7-bit
bytes (ie, as if you were going to transmit it over midi in a SYSEX message). Of course, you
will have a variable number of bytes depending upon your delta-time. To indicate which is the
last byte of the series, you leave bit #7 clear. In all of the preceding bytes, you set bit #7. So,
if a delta-time is between 0-127, it can be represented as one byte. The largest delta-time
allowed is 0FFFFFFF, which translates to 4 bytes variable length. Here are examples of
delta-times as 32-bit values, and the variable length quantities that they translate to:

```
NUMBER        VARIABLE QUANTITY
 00000000              00
 00000040              40
 0000007F              7F
 00000080             81 00
 00002000             C0 00
 00003FFF             FF 7F
 00004000           81 80 00
 00100000           C0 80 00
 001FFFFF           FF FF 7F
 00200000          81 80 80 00
 08000000          C0 80 80 00
 0FFFFFFF          FF FF FF 7F
```

Here's some C routines to read and write variable length quantities such as delta-times. With
**WriteVarLen()**, you pass a 32-bit value (ie, unsigned long) and it spits out the correct
series of bytes to a file. **ReadVarLen()** reads a series of bytes from a file until it
reaches the last byte of a variable length quantity, and returns a 32-bit value.

```
void WriteVarLen(register unsigned long value)
{
   register unsigned long buffer;
   buffer = value & 0x7F;

   while ( (value >>= 7) )
   {
     buffer <<= 8;
     buffer |= ((value & 0x7F) | 0x80);
   }

   while (TRUE)
   {
      putc(buffer,outfile);
      if (buffer & 0x80)
          buffer >>= 8;
      else
          break;
   }
}


doubleword ReadVarLen()
{
    register doubleword value;
    register byte c;

    if ( (value = getc(infile)) & 0x80 )
    {
       value &= 0x7F;
       do
       {
         value = (value << 7) + ((c = getc(infile)) & 0x7F);
       } while (c & 0x80);
    }

    return(value);
}
```

**NOTE:** The concept of variable length quantities (ie, breaking up a large value into a series
of bytes) is used with other fields in a MIDI file besides delta-times, as you'll see later.

## Events

The first (1 to 4) byte(s) in an MTrk will be the first event's delta-time as a variable length
quantity. The next data byte is actually the first byte of that event itself. I'll refer to this as
the event's *Status*. For MIDI events, this will be the actual MIDI Status byte
(or the first midi data byte if running status). For example, if the byte is hex 90, then this event
is a *Note-On* upon midi channel 0. If for example, the byte was hex 23, you'd have to
recall the previous event's status (ie, midi running status). Obviously, the first MIDI event in the
MTrk **must** have a status byte. After a midi status byte comes its 1 or 2 data bytes
(depending upon the status - some MIDI messages only have 1 subsequent data byte). After that
you'll find the next event's delta time (as a variable quantity) and start the process of reading that
next event.

SYSEX (system exclusive) events (status = F0) are a special case because a SYSEX event can
be any length. After the F0 status (which is always stored -- no running status here), you'll find
yet another series of variable length bytes. Combine them with ReadVarLen() and you'll come up
with a 32-bit value that tells you how many more bytes follow which make up this SYSEX event.
This length doesn't include the F0 status.

For example, consider the following SYSEX MIDI message:

F0 7F 7F 04 01 7F 7F F7

This would be stored in a MIDI file as the following series of bytes (minus the delta-time
bytes which would precede it):

F0 07 7F 7F 04 01 7F 7F F7

Some midi units send a system exclusive message as a series of small "packets" (with a time
delay inbetween transmission of each packet). The first packet begins with an F0, but it doesn't
end with an F7. The subsequent packets don't start with an F0 nor end with F7. The last packet
doesn't start with an F0, but does end with the F7. So, between the first packet's opening F0 and
the last packet's closing F7, there's 1 SYSEX message there. (Note: only extremely poor designs,
such as the crap marketed by Casio exhibit these aberrations). Of course, since a delay is needed
inbetween each packet, you need to store each packet as a separate event with its own time in the
MTrk. Also, you need some way of knowing which events shouldn't begin with an F0 (ie, all of
them except the first packet). So, the MIDI file spec redefines a midi status of F7 (normally used
as an end mark for SYSEX packets) as a way to indicate an event that doesn't begin with F0. If
such an event follows an F0 event, then it's assumed that the F7 event is the second "packet" of
a series. In this context, it's referred to as a SYSEX CONTINUATION event. Just like the F0 type
of event, it has a variable length followed by data bytes. On the other hand, the F7 event could be
used to store MIDI REALTIME or MIDI COMMON messages. In this case, after the variable length
bytes, you should expect to find a MIDI Status byte of F1, F2, F3, F6, F8, FA, FB, FC, or FE. (Note
that you wouldn't find any such bytes inside of a SYSEX CONTINUATION event). When used in this
manner, the F7 event is referred to as an ESCAPED event.

A status of FF is reserved to indicate a special non-MIDI event. (Note that FF is used in MIDI
to mean "reset", so it wouldn't be all that useful to store in a data file. Therefore, the MIDI file
spec arbitrarily redefines the use of this status). After the FF status byte is another byte that
tells you what *Type* of non-MIDI event it is. It's sort of like a second status byte. Then
after this byte is another byte(s -- a variable length quantity again) that tells how many more data
bytes follow in this event (ie, its Length). This Length doesn't include the FF, Type
byte, nor the Length byte. These special, non-MIDI events are called *Meta-Events*, and
most are optional unless otherwise noted. What follows are some defined Meta-Events (including
the FF Status and Length). Note that unless otherwise mentioned, more than one of these events
can be placed in an Mtrk (even the same Meta-Event) at any delta-time. (Just like all midi
events, Meta-Events have a delta-time from the previous event regardless of what type of event
that may be. So, you can freely intermix MIDI and Meta events).

### Sequence Number

FF 00 02 ss ss

This optional event which must occur at the beginning of a MTrk (ie, before any non-zero
delta-times and before any midi events) specifies the number of a sequence. The two data bytes
**ss ss**, are that number which corresponds to the *MIDI Cue* message. In a
format 2 MIDI file, this number identifies each "pattern" (ie, Mtrk) so that a "song" sequence
can use the MIDI Cue message to refer to patterns. If the **ss ss** numbers are omitted
(ie, Length byte = 0 instead of 2), then the MTrk's location in the file is used (ie, the
first MTrk chunk is the first pattern). In format 0 or 1, which contain only one "pattern" (even
though format 1 contains several MTrks), this event is placed in only the first MTrk. So, a group
of format 1 files with different sequence numbers can comprise a "song collection".

There can be only one of these events per MTrk chunk in a Format 2. There can be only one
of these events in a Format 0 or 1, and it must be in the first MTrk.

### Text

FF 01 len text

Any amount of text (amount of bytes = **len**) for any purpose. It's best to put this
event at the beginning of an MTrk. Although this text could be used for any purpose, there are
other text-based Meta-Events for such things as orchestration, lyrics, track name, etc. This
event is primarily used to add "comments" to a MIDI file which a program would be expected to
ignore when loading that file.

Note that **len** could be a series of bytes since it is expressed as a variable length
quantity.

### Copyright

FF 02 len text

A copyright message (ie, text). It's best to put this event at the beginning of an MTrk.

Note that **len** could be a series of bytes since it is expressed as a variable length
quantity.

### Sequence/Track Name

FF 03 len text

The name of the sequence or track (ie, text). It's best to put this event at the beginning of an
MTrk.

Note that **len** could be a series of bytes since it is expressed as a variable length
quantity.

### Instrument

FF 04 len text

The name of the instrument that the track plays (ie, text). This might be different than the
Sequence/Track Name. For example, maybe the name of your sequence (ie, Mtrk) is "Butterfly",
but since the track is played on a piano, you might also include an Instrument Name of "Piano".

It's best to put one (or more) of this event at the beginning of an MTrk to provide the user with
identification of what instrument(s) is playing the track. Usually, the instruments (ie, patches,
tones, banks, etc) are setup on the audio devices via *MIDI Program Change* events
within the MTrk, particularly in MIDI files that are intended for *General MIDI Sound
Modules*. So, this event exists merely to provide the user with visual feedback of the
instrumentation for a track.

Note that **len** could be a series of bytes since it is expressed as a variable length
quantity.

### Lyric

FF 05 len text

A song lyric (ie, text) which occurs on a given beat. A single Lyric
MetaEvent should contain only one syllable.

Note that **len** could be a series of bytes since it is expressed as a variable length
quantity.

### Marker

FF 06 len text

A marker (ie, text) which occurs on a given beat. Marker events might be used to denote a loop
start and loop end (ie, where the sequence loops back to a previous event).

Note that **len** could be a series of bytes since it is expressed as a variable length
quantity.

### Cue Point

FF 07 len text

A cue point (ie, text) which occurs on a given beat. A Cue Point might be used to denote
where a WAVE (ie, sampled sound) file starts playing, where the **text** would be the
WAVE's filename.

Note that **len** could be a series of bytes since it is expressed as a variable length
quantity.

### MIDI Channel

FF 20 01 cc

This optional event which normally occurs at the beginning of an MTrk (ie, before any non-zero
delta-times and before any MetaEvents except Sequence Number) specifies to which MIDI Channel any subsequent
MetaEvent or System Exclusive events are associated. The data byte **cc**, is the MIDI channel, where 0 would be the
first channel.

The MIDI spec does not give a MIDI channel to System Exclusive events. Nor do MetaEvents have an imbedded channel.
When creating a Format 0 MIDI file, all of the System Exclusive and MetaEvents go into one track, so its hard to associate
these events with respective MIDI Voice messages. (ie, For example, if you wanted to name the musical part on MIDI channel
1 "Flute Solo", and the part on MIDI Channel 2 "Trumpet Solo", you'd need to use 2 Track Name MetaEvents. Since both events
would be in the one track of a Format 0 file, in order to distinguish which track name was associated with which MIDI
channel, you would place a MIDI Channel MetaEvent with a channel number of 0 before the "Flute Solo" Track Name MetaEvent,
and then place another MIDI Channel MetaEvent with a channel number of 1 before the "Trumpet Solo" Track Name MetaEvent.

It is acceptable to have more than one MIDI channel event in a given track, if that track needs to associate various
events with various channels.

### MIDI Port

FF 21 01 pp

This optional event which normally occurs at the beginning of an MTrk (ie, before any non-zero
delta-times and before any midi events) specifies out of which MIDI Port (ie, buss) the MIDI events in the
MTrk go. The data byte **pp**, is the port number, where 0 would be the first MIDI buss in the
system.

The MIDI spec has a limit of 16 MIDI channels per MIDI input/output (ie, port, buss, jack, or whatever terminology
you use to describe the hardware for a single MIDI input/output). The MIDI channel number for a given event is
encoded into the lowest 4 bits of the event's Status byte. Therefore, the channel number is always 0 to
15. Many MIDI interfaces have multiple MIDI input/output busses in order to work around limitations in the MIDI
bandwidth (ie, allow the MIDI data to be sent/received more efficiently to/from several external modules), and to
give the musician more than 16 MIDI Channels. Also, some sequencers support more than one MIDI interface
used for simultaneous input/output. Unfortunately, there is no way to encode more than 16 MIDI channels
into a MIDI status byte, so a method was needed to identify events that would be output on, for example,
channel 1 of the second MIDI port versus channel 1 of the first MIDI port. This MetaEvent allows a sequencer
to identify which MTrk events get sent out of which MIDI port. The MIDI events following a MIDI Port MetaEvent
get sent out that specified port.

It is acceptable to have more than one Port event in a given track, if that track needs to output to another port at
some point in the track.

### End of Track

FF 2F 00

This event is NOT optional. It must be the last event in every MTrk. It's used as a definitive
marking of the end of an MTrk. Only 1 per MTrk.

### Tempo

FF 51 03 tt tt tt

Indicates a tempo change. The 3 data bytes of **tt tt tt** are the tempo in microseconds per quarter note. In other
words, the microsecond tempo value tells you how long each one of your sequencer's "quarter notes" should be. For example,
if you have the 3 bytes of 07 A1 20, then each quarter note should be 0x07A120 (or 500,000) microseconds long.

So, the MIDI file format expresses tempo as "the amount of time (ie, microseconds) per quarter note".

#### BPM

Normally, musicians express tempo as "the amount of quarter notes in every minute (ie, time period)". This is the opposite
of the way that the MIDI file format expresses it.

When musicians refer to a "beat" in terms of tempo, they are referring to a quarter note (ie, a quarter note is always 1
beat when talking about tempo, regardless of the time signature. Yes, it's a bit confusing to non-musicians that the
time signature's "beat" may not be the same thing as the tempo's "beat" -- it won't be unless the time signature's beat also
happens to be a quarter note. But that's the traditional definition of BPM tempo). To a musician, tempo is therefore always
"how many quarter notes happen during every minute". Musicians refer to this measurement as BPM (ie, Beats Per Minute). So a
tempo of 100 BPM means that a musician must be able to play 100 steady quarter notes, one right after the other, in one
minute. That's how "fast" the "musical tempo" is at 100 BPM. It's very important that you understand the concept of how a
musician expresses "musical tempo" (ie, BPM) in order to properly present tempo settings to a musician, and yet be able to
relate it to how the MIDI file format expresses tempo.

To convert the MIDI file format's tempo (ie, the 3 bytes that specify the amount of microseconds per quarter note) to
BPM:

BPM = 60,000,000/(tt tt tt)

For example, a tempo of 120 BPM = 07 A1 20.

So why does the MIDI file format use "time per quarter note" instead of "quarter notes per time" to specify its tempo?
Well, its easier to specify more precise tempos with the former. With BPM, sometimes you have to deal with fractional tempos
(for example, 100.3 BPM) if you want to allow a finer resolution to the tempo. Using microseconds to express tempo offers
plenty of resolution.

Also, SMPTE is a time-based protocol (ie, it's based upon seconds, minutes, and hours, rather than a musical tempo).
Therefore it's easier to relate the MIDI file's tempo to SMPTE timing if you express it as microseconds. Many musical
devices now use SMPTE to sync their playback.

#### PPQN Clock

A sequencer typically uses some internal hardware timer counting off steady time (ie, microseconds perhaps) to generate a
software "PPQN clock" that counts off the timebase (Division) "ticks". In this way, the time upon which an event occurs can
be expressed to the musician in terms of a musical bar:beat:PPQN-tick rather than how many microseconds from the start of the
playback. Remember that musicians always think in terms of a beat, not the passage of seconds, minutes, etc.

As mentioned, the microsecond tempo value tells you how long each one of your sequencer's "quarter notes" should be. From
here, you can figure out how long each one of your sequencer's PPQN clocks should be by dividing that microsecond value by
your MIDI file's Division. For example, if your MIDI file's Division is 96 PPQN, then that means that each of your
sequencer's PPQN clock ticks at the above tempo should be 500,000 / 96 (or 5,208.3) microseconds long (ie, there should be
5,208.3 microseconds in between each PPQN clock tick in order to yield a tempo of 120 BPM at 96 PPQN. And there should
always be 96 of these clock ticks in each quarter note, 48 ticks in each eighth note, 24 ticks in each sixteenth, etc).

Note that you can have any timebase at any tempo. For example, you can have a 96 PPQN file playing at
100 BPM just as you can have a 192 PPQN file playing at 100 BPM. You can also have a 96 PPQN file playing at either 100
BPM or 120 BPM. Timebase and tempo are two entirely separate quantities. Of course, they both are needed when you setup your
hardware timer (ie, when you set how many microseconds are in each PPQN tick). And of course, at slower tempos, your
PPQN clock tick is going to be longer than at faster tempos.

#### MIDI Clock

MIDI clock bytes are sent over MIDI, in order to sync the playback of 2 devices (ie, one device is generating MIDI clocks at
its current tempo which it internally counts off, and the other device is syncing its playback to the receipt of these
bytes). Unlike with SMPTE frames, MIDI clock bytes are sent at a rate related to the musical tempo.

Since there are 24 MIDI Clocks in every quarter note, the length of a MIDI Clock (ie, time inbetween each MIDI
Clock message) is the microsecond tempo divided by 24. In the above example, that would be 500,000/24, or 20,833.3
microseconds in every MIDI Clock. Alternately, you can relate this to your timebase (ie, PPQN clock). If you have 96 PPQN,
then that means that a MIDI Clock byte must occur every 96 / 24 (ie, 4) PPQN clocks.

#### SMPTE

SMPTE counts off the passage of time in terms of seconds, minutes, and hours (ie, the way that non-musicians count time).
It also breaks down the seconds into smaller units called "frames". The movie industry created SMPTE, and they adopted 4
different frame rates. You can divide a second into 24, 25, 29, or 30 frames. Later on, even finer resolution was needed by
musical devices, and so each frame was broken down into "subframes".

So, SMPTE is not directly related to musical tempo. SMPTE time doesn't vary with "musical tempo".

Many devices use SMPTE to sync their playback. If you need to sychronize with such a device, then you may need to deal
with SMPTE timing. Of course, you're probably still going to have to maintain some sort of PPQN clock, based upon the
passing SMPTE subframes, so that the user can adjust the tempo of the playback in terms of BPM, and can consider the time
of each event in terms of bar:beat:tick. But since SMPTE doesn't directly relate to musical tempo, you have to interpolate
(ie, calculate) your PPQN clocks from the passing of subframes/frames/seconds/minutes/hours (just as we previously
calculated the PPQN clock from a hardware timer counting off microseconds).

Let's take the easy example of 25 Frames and 40 SubFrames. As previously mentioned in the discussion of Division, this
is analogous to millisecond based timing because you have 1,000 SMPTE subframes per second. (You have 25 frames per
second. Each second is divided up into 40 subframes, and you therefore have 25 \* 40 subframes per second. And remember that
1,000 milliseconds are also in every second). Every millisecond therefore means that another subframe has passed (and vice
versa). Every time you count off 40 subframes, a SMPTE frame has passed (and vice versa). Etc.

Let's assume you desire 96 PPQN and a tempo of 500,000 microseconds. Considering that with 25-40
Frame-SubFrame SMPTE timing 1 millisecond = 1 subframe (and remember that 1 millisecond = 1,000 microseconds), there
should be 500,000 / 1,000 (ie, 500) subframes per quarter note. Since you have 96 PPQN in every quarter note, then every
PPQN ends up being 500 / 96 subframes long, or 5.2083 milliseconds (ie, there's how we end up with that 5,208.3 microseconds
PPQN clock tick just as we did above in discussing PPQN clock). And since 1 millisecond = 1 subframe, every PPQN clock
tick also equals 5.2083 subframes at the above tempo and timebase.

#### Conclusions

BPM = 60,000,000/MicroTempo
  
MicrosPerPPQN = MicroTempo/TimeBase
  
MicrosPerMIDIClock = MicroTempo/24
  
PPQNPerMIDIClock = TimeBase/24
  
MicrosPerSubFrame = 1000000 \* Frames \* SubFrames
  
SubFramesPerQuarterNote = MicroTempo/(Frames \* SubFrames)
  
SubFramesPerPPQN = SubFramesPerQuarterNote/TimeBase
  
MicrosPerPPQN = SubFramesPerPPQN \* Frames \* SubFrames

### SMPTE Offset

FF 54 05 hr mn se fr ff

Designates the SMPTE start time (hours, minutes, secs, frames, subframes) of the MTrk. It should
be at the start of the MTrk. The hour should not be encoded with the SMPTE format as it is in
*MIDI Time Code*. In a format 1 file, the SMPTE OFFSET must be stored with the tempo
map (ie, the first MTrk), and has no meaning in any other MTrk. The **ff** field contains
fractional frames in 100ths of a frame, even in SMPTE based MTrks which specify a different
frame subdivision for delta-times (ie, different from the subframe setting in the MThd).

### Time Signature

FF 58 04 nn dd cc bb

Time signature is expressed as 4 numbers. **nn** and **dd** represent the
"numerator" and "denominator" of the signature as notated on sheet music. The denominator is a
negative power of 2: 2 = quarter note, 3 = eighth, etc. The **cc** expresses the number of
MIDI clocks in a metronome click. The **bb** parameter expresses the number of notated
32nd notes in a MIDI quarter note (24 MIDI clocks). This event allows a program to relate what
MIDI thinks of as a quarter, to something entirely different. For example, 6/8 time with a
metronome click every 3 eighth notes and 24 clocks per quarter note would be the following
event:

FF 58 04 06 03 18 08

### Key Signature

FF 59 02 sf mi

**sf** = -7 for 7 flats, -1 for 1 flat, etc, 0 for key of c, 1 for 1 sharp, etc.

**mi** = 0 for major, 1 for minor

### Proprietary Event

FF 7F len data...

This can be used by a program to store proprietary data. The first byte(s) should be a unique ID
of some sort so that a program can identity whether the event belongs to it, or to some other
program. A 4 character (ie, ascii) ID is recommended for such.

Note that **len** could be a series of bytes since it is expressed as a variable length
quantity.

---

## Errata

In a format 0 file, the tempo and time signature changes are scattered throughout the one
MTrk. In format 1, the very first MTrk should consist of just the tempo and time signature events
so that it could be read by some device capable of generating a "tempo map". In format 2, each
MTrk should begin with at least one initial tempo and time signature event.

**NOTE:** If there are no tempo and time signature events in a MIDI file, assume 120 BPM and 4/4.

## RMID Files

The method of saving data in chunks (ie, where the data is preceded by
an 8 byte header consisting of a 4 char ID and a 32-bit size field) is the
basis for Interchange File Format. You should now read the article
About Interchange File Format for background
information.

As mentioned, MIDI File format is a "broken" IFF. It lacks a file header
at the start of the file. One bad thing about this is that a standard IFF
parsing routine will choke on a MIDI file (because it will expect the first
12 bytes to be the group ID, filesize, and type ID fields). In order to
fix the MIDI File format so that it strictly adheres to IFF, Microsoft
simply made up a 12-byte header that is prepended to MIDI files, and thereby
came up with the RMID format. An RMID file begins with the group ID (4
ascii chars) of 'R', 'I', 'F', 'F', followed by the 32-bit filesize field,
and then the type ID of 'R', 'M', 'I', 'D'. Then, the chunks of a MIDI
file follow (ie, the MThd and MTrk chunks). If you chop off the first 12
bytes of an RMID file, then you end up with a standard MIDI file.

Note that chunks within a MIDI file are **not** padded out (with an
extra 0 byte) to an even number of bytes. I don't know as if the RMID
format corrects this aberration of the MIDI file format too.
