# Supplementary material for: An auditory display tool for DNA sequence analysis
Source: BMC Bioinformatics. 2017 Apr 24;18:221. doi: 10.1186/s12859-017-1632-x (PMC5404335; doi:10.1186/s12859-017-1632-x)
Supplement: Supplementary file 17 — Code for website; including html, php and associated files. (ZIP 49453 kb) [file 12859_2017_1632_MOESM17_ESM.zip › sonification/midi_class_v175/readme.htm]

PHP MIDI CLASS


# PHP MIDI CLASS

## Description

Class for reading, writing, analyzing, modifying, creating, downloading
and playing (embedding) standard MIDI files (\*.mid, \*.rmi) of type 0 or 1. MIDI songs are
internally represented as lists of tracks, where each track is a list of
messages, and each message is a string. The message string format is
the same as the one used by the commandline tools MF2T/T2MF
created by Piet van Oostrum.  
  
The class provides methods to generate and manipulate MIDI data and to import and export binary midi files (SMF, \*.mid), text in the MF2T/T2MF format and MIDI XML.  

## Applications

- audio toys
- mixers
- sequencers
- ringtone creators
- musical education/training
- ...

## Documentation

## Demos

manipulate.php  
demonstrates manipulation of MIDI data (imported MIDI file) in various
ways
  
  
sequencer.php  
little online sequencer, 4 drum tracks, 4 instrument tracks, 1 bar
only, result can be saved (simple mix format: serialized post array).
  
  
mid2txt.php  
demonstrates binary MIDI file to text (MF2T/T2MF format) conversion
  
  
txt2mid.php  
demonstrates text (MF2T/T2MF format) to binary MIDI file conversion
  
  
mid2xml.php  
demonstrates binary MIDI file to MIDI XML conversion
  
  
xml2mid.php  
demonstrates MIDI XML to binary MIDI file conversion
  
  
meta.php  
shows content of all meta events in the first track of a MIDI file. These events are often used for song title, copyright informations etc. (like ID3 tags in mp3 files).
  
  
duration.php  
demonstrates how to find the duration of a MIDI file. uses a simple child class.
  
  
convert.php  
converts MIDI files of type 1 to type 0. uses a simple child class.
  
  
volume.php  
demonstrates changing the (channel) volume(s). uses a simple child class.
  
  
trim.php  
demonstrates trimming a midi file. uses a simple child class.
  
  
mid2rttl.php  
demonstrates (simple) binary MIDI file to RTTL ringtone conversion (uses the RTTL extension, see downloads).
  
  
rttl2mid.php  
demonstrates RTTL ringtone to binary MIDI file conversion (uses the RTTL extension, see downloads).
  
  

## Downloads

## Related Links

MIDI Manufacturers Association (MMA)  
MMA's MIDI XML Specifications  
Recordare  
Standard MIDI File DTD: MIDI XML  
MF2T/T2MF  
MIDI-OX  
Beatnik

## Contact

fluxus at freenet dot de
  
  
