# Supplementary material for: An auditory display tool for DNA sequence analysis
Source: BMC Bioinformatics. 2017 Apr 24;18:221. doi: 10.1186/s12859-017-1632-x (PMC5404335; doi:10.1186/s12859-017-1632-x)
Supplement: Supplementary file 17 — Code for website; including html, php and associated files. (ZIP 49453 kb) [file 12859_2017_1632_MOESM17_ESM.zip › sonification/midi_class_v175/tmp/testPlayMIDI.php]

playMIDI MIDI.js test

  
Play DNA audio.mid file
  
This file plays fine
  
  
Play 14673430.mid file
  
This file does not play
  
  
Stop MIDI Playback
